# Study protocol for improving asthma outcomes through cross-cultural communication training for physicians: a randomized trial of physician training

**DOI:** 10.1186/1472-6920-14-118

**Published:** 2014-06-16

**Authors:** Minal R Patel, Lara J Thomas, Kausar Hafeez, Matthew Shankin, Margaret Wilkin, Randall W Brown

**Affiliations:** 1 Center for Managing Chronic Disease, 1415 Washington Heights, Ann Arbor, MI 48109-2029, USA; 2 Department of Health Behavior and Health Education, University of Michigan School of Public Health, 1415 Washington Heights, Ann Arbor, MI 48109-2029, USA

**Keywords:** Asthma, Randomized controlled trials, Physicians, Communication, Health disparities

## Abstract

**Background:**

Massive resources are expended every year on cross-cultural communication training for physicians. Such training is a focus of continuing medical education nationwide and is part of the curriculum of virtually every medical school in America. There is a pressing need for evidence regarding the effects on patients of cross-cultural communication training for physicians. There is a need to understand the added benefit of such training compared to more general communication. We know of no rigorous study that has assessed whether cross-cultural communication training for physicians results in better health outcomes for their patients. The current study aims to answer this question by enhancing the Physician Asthma Care Education (PACE) program to cross cultural communication (PACE Plus), and comparing the effect of the enhanced program to PACE on the health outcomes of African American and Latino/Hispanic children with asthma.

**Methods/Design:**

A three-arm randomized control trial is used to compare PACE Plus, PACE, and usual care. Both PACE and PACE Plus are delivered in two, two-hour sessions over a period of two weeks to 5–10 primary care physicians who treat African American and Latino/Hispanic children with asthma. One hundred twelve physicians and 1060 of their pediatric patients were recruited who self-identify as African American or Latino/Hispanic and experience persistent asthma. Physicians were randomized into receiving either the PACE Plus or PACE intervention or into the control group. The comparative effectiveness of PACE and PACE Plus on clinician’s therapeutic and communication practices with the family/patient, children’s urgent care use for asthma, asthma control, and quality of life, and parent/caretaker satisfaction with physician performance will be assessed. Data are collected via telephone survey and medical record review at baseline, 9 months following the intervention, and 21 months following the intervention.

**Discussion:**

This study aims to reduce disparities in asthma outcomes among African American and Latino/Hispanic children through cross-cultural communication training of their physicians and assessing the added value of this training compared to general communication. The results of this study will provide important information about the value of cross-cultural training in helping to address persistent racial disparities in outcomes.

**Trial registration:**

ClinicalTrials.gov: NCT01251523 December 1, 2010

## Background

Asthma affects 7.1 million U.S. children and is the main reason children use urgent care services for a chronic disease [1]. In 2009, 1 in 5 children with asthma sought care in an emergency department [2]. Asthma prevalence among children and adolescents is estimated to be 9.6% overall, and is highest among non-Hispanic black children (17.0%) [2]. Hispanic/Latino and non-Hispanic black children have greater asthma-related risk exposures than non-Hispanic Whites, and are at higher risk for emergency department visits, hospitalizations, and death from asthma [3-6]. They exhibit lower adherence to medication and only about one-third report using long-term controller medicine or receiving asthma care plans from their clinicians [2]. Poor outcomes are especially striking among African American children, who have a 260% higher emergency department (ED) visit rate, a 250% higher hospitalization rate, and a 500% higher death rate from asthma than their White counterparts [7]. Because the risk, severity, and control of asthma are all influenced by a combination of genetic, social, and environmental factors, reducing the impact of the condition has proven difficult. However, enhancing clinical care in populations with marked disparities is regarded as a priority effort [8,9]. Asthma is most often managed in the primary care setting and 80% of those affected never visit an asthma specialist [2]. Given the high prevalence of asthma in children and the associated frequency of visits to primary care practitioners, these health care providers are the asthma patients’ primary source of care and education. Thus improving asthma clinical care in primary care providers offers the potential to reduce the burden of asthma in underserved populations.

The uncertainty of physicians in their interactions with minority patients, the clinicians’ beliefs or stereotypes regarding the health behavior of minority patients, and patient response to perceived negative experiences with providers all have been discussed extensively in the literature [10]. The dramatic demographic shifts occurring in the United States mean that increasing numbers of minority patients are being seen in the health care system and, for the foreseeable future, the majority of the physicians who treat them are unlikely to be of their same racial or ethnic background. Further, it is increasingly expected that health care institutions account for the social and cultural diversity of the U.S. population [11] especially as it relates to clinical practice, and performance expectations for practicing physicians are also very high.

Massive resources are expended every year on cross-cultural communication training for physicians. Such training is a focus of continuing medical education nationwide and is part of the curriculum of virtually every medical school in America. There is a pressing need for evidence regarding the effects on patients of cross-cultural communication training for physicians. There is a need to understand the added benefit of such training compared to training in more general communication and patient-physician partnership skills [12]. However, we know of no rigorous study that has assessed whether cross-cultural communication training for physicians results in better health outcomes for their patients.

### Adapting the Physician Asthma Care Education Program

The Physician Asthma Care Education (PACE) Program is a two-part interactive, multi-media educational seminar developed two-decades ago and widely disseminated to improve physician awareness, ability, and use of communication and therapeutic techniques for reducing the effects of asthma on children and their families. Efficacy and effectiveness trials have shown that patients of PACE-trained physicians demonstrate improvements in long-term outcomes including fewer symptoms and less health care use [13,14]. Importantly, no more time is required in the patient-physician encounter by PACE-trained physicians. This general communication intervention provides an effective comparison to investigate the added value of training in cross-cultural communication. PACE was enhanced to focus on cross-cultural communication training. This enhanced intervention, PACE Plus, was developed with cultural anthropology experts and piloted on three occasions with 31 primary care physicians. Unpublished data reveal that the majority of participants believed the training to be very useful and relevant, and participants demonstrated significant improvement in their understanding of cross-cultural communication, and increased confidence in engaging in tailored communication with underserved populations.

## Methods/Design

This study used a randomized controlled design with three arms: control, the original PACE intervention, and PACE Plus intervention (enhanced with cross-cultural communication training) to test the comparative effectiveness of PACE with cross-cultural communication training, a proven asthma-based communication intervention for physicians. All study procedures were reviewed and approved by the University of Michigan Health Sciences and Behavioral Sciences Institutional Review Board (IRB-HSBS HUM#00030087).

### Study hypotheses

The study addressed two questions: 1) Does cross-cultural communication training (PACE Plus) produce better outcomes for African American and Latino/Hispanic children with asthma, and their respective parents, than a general communication training program (PACE)?; 2) Compared to the control group, is PACE, already shown to be effective with the general population of patients, effective when used specifically with African American and Latino/Hispanic children with asthma, and their parents?

The study hypothesis is that there will be positive outcomes for patients of physicians in both interventions but better outcomes for those patients whose doctors participate in the cross-cultural communication training (PACE Plus). That is, PACE Plus compared to PACE will result in reductions in children’s health care use for asthma, improved symptom experience for the children, greater parent/caretaker satisfaction with the physician’s performance, enhanced asthma related quality of life for parents/caretakers of the patients, higher levels of confidence and value placed by physicians on skills needed when working cross culturally, and increased use of National Asthma Education and Prevention Program (NAEPP) recommended therapies by physicians. It is also hypothesized that PACE compared to a control group will produce better outcomes on the aforementioned, six dimensions.

### Sample size determination

The sample size of 1002 children and their parents total at baseline was determined by power calculations using the primary outcome of proportion of subjects with emergency department (ED) visits in the last twelve months taking into account a 25% attrition rate over 24 months leaving 801 total subjects at the end of the study. The sample size calculation is based on the assumption that the clustering of patients within physician practices is negligible, which is justified from the previous PACE study [14]. In that prior research, the intracluster correlation values were close to zero (0.24 at the physician level; 0.003 at the site level). In analysis, such correlation will be adjusted for, if necessary. In the previous PACE study [14], the baseline proportion of patient participants with an ED visit was 30% and this was decreased by 15% in 24 months. For the current study, there will be 80% power to detect a more conservative difference of 12% or more in the proportion of patient participants with an ED visit in 24 months at the significance level of 0.05.

In the previous PACE study, those in the intervention group decreased by a little more than one mean emergency office visit and 0.09 mean hospitalizations [14]. With the sample size in the current study, there will be statistical power to detect changes at that level for hospitalization and at much lower levels than in the previous PACE study for emergency office visits. Given the relatively rare occurrence of emergency department visits, the sample size needed to detect changes is relatively large; this gives an advantage in having sufficient statistical power to detect small changes in other outcomes, including symptoms, patient satisfaction and asthma related quality of life.

### Physician and parent recruitment

#### *Physicians*

A total of 112 physicians were recruited in Atlanta, GA, the Bronx, NY and several communities in Michigan. To be eligible, physicians met the inclusion criteria outlined in Table 1. The sites chosen represented areas with a large African American and Hispanic/Latino populations being served by physicians that *did not* self-identify as African American and Hispanic/Latino.

**Table 1:** Inclusion criteria for participation in current RCT

**Physician criteria**	**Patient criteria**	**Parent criteria**
1. Licensed physician in practice - board certified pediatrics/family medicine	1. Treated by the participating physician during the study intake period	1. Has primary responsibility for the child’s care
2. Treating children with asthma	2. Age 1–16 years	2. Self-identified as African American or Hispanic/Latino
3. Full-time (>25 hrs.) in a practice in GA, MI or NY	3. Diagnosis of asthma by a physician using NAEPP Guidelines (alternative descriptions such as reactive airway disease, bronchitis, or wheezy bronchitis were not accepted)	3. Has access to a telephone
4. Not Hispanic/Latino or African American in ethnicity/race (this allowed us to test the PACE Plus components that addressed cross cultural communication)	4. Has persistent asthma as defined by NAEPP classification for asthma severity	4. Consents to participate
5. Consented to participate	5. Does not have other chronic disorders that cause pulmonary complications, e.g. sickle cell disease	
6. Willing to generate a roster of pediatric African American and Hispanic/Latino asthma patients	6. Self-identify as African American or Hispanic/Latino	

To recruit physicians, a roster of potential physician participants was generated using membership lists from, e.g., local professional societies, asthma coalitions, and yellow-pages listings. Recruitment packets were initially mailed to potential candidates. The packet included a cover letter from the local physician co-investigator, a brochure describing the study, a brief screening form and questionnaire with a self-addressed, stamped envelope. Seven to ten days after the mailing, the recruitment coordinator called each physician office, spoke to a practice manager and arranged a day and time for the physician co-investigator to speak directly with the potential physician recruit. Personal contact by a local clinical practice leader was an important strategy for recruiting physicians [15-17]. Multiple visits and phone calls to physician offices and practice managers were not uncommon due to busy clinic schedules.

Physicians were told that participation involved generating a roster of their patients with asthma, completing 3 self-administered interviews over 2 years, and willingness to be randomly assigned to one of three study conditions. Every effort was made to minimize physician or office staff burden. For example, research-related meetings with office staff were kept to a minimum. All enrolled physicians received, a $50 gift card for completing each of the three data collection questionnaires and $50 for their office for providing a patient list in Year 1 and health care use data at the end of the study. Physicians randomized to either the PACE or PACE Plus interventions arms received 5 CME credits for completing the intervention sessions.

#### *Child and parent recruitment*

A total of 1,060 patients were recruited from the pool of patients provided by enrolled physicians. To be eligible, the child needed to meet the inclusion criteria listed in Table 1 and their parent or caregiver needed to meet the eligibility criteria as well.

Initially, the office staff of a participating physician was asked to provide a list of patients who met the eligibility criteria using office records. The patient list was provided to research staff. Eligible patients/caretakers were then mailed a recruitment packet containing a cover letter from the participating physician inviting them to enroll, a study overview page, two copies of the consent form, a health information screener form, a decline postcard, and a stamped self-addressed envelope for returning a copy of the consent and screener form.

Following the mailed information, patients received recruitment phone calls, which provided information about the study’s purpose, design, randomization, and data collection. Calls were made by trained bilingual staff, to ensure the same information was provided to all potential participants. Participants could opt to complete the consent and screener via paper and mail them back, via telephone with a trained staff member. Once consent was received by the research staff, parents were contacted by a telephone interviewer and scheduled for a baseline interview. The recruitment scripts, consent forms, and the telephone interviews were available in both English and Spanish. Children/parents subsequently followed physicians into one of the three study arms as randomized. Parents were not informed if their physician was participating in the intervention.

The following ongoing strategies were employed to enhance recruitment and retention. 1) caretakers received a $30 gift card following completion of the baseline data-collection interview and a $20 gift card for each subsequent completed follow-up interview; 2) Mother’s and Father’s Day cards were mailed annually to maintain a personal connection; 3) a study newsletter, mailed bi-annually, provided updates on study progress and basic asthma information; 4) repeated attempts were made over a year to check for reconnected phone lines; 5) the study team maintained contact with physician offices and periodically asked for updated contact information for enrolled participants.

### Randomization

#### *Experimental design and randomization*

A randomized controlled design was used in this study. At the beginning of the study, a permuted block randomization schedule was created using the RAN TBL and RANUNI functions in SAS to generate blocks of two for random assignment of physicians. Data are collected from three sites, Atlanta, Georgia, New York City, New York, and cities throughout Michigan. It is recognized that geographic site as well as size of practice of the physicians selected for study could impact on results. Thus, randomization was stratified by geographic area (three levels, Atlanta area, metropolitan New York, and Michigan) and size of practice (two levels, solo practice and multiple-physician practices). This randomization ensured that each type of practice and geographic area would be evenly distributed across the three arms of the study, which reduces bias due to these potentially confounding factors in the comparative analysis. Once physicians consented to participate, they were placed into one of the three arms of the study according to the randomization schedule with 44 physicians assigned to PACE, 36 to PACE PLUS and 32 to control (see sample size determination) with a total of 112 physicians and 1062 patients following those physicians into randomization.

Because this evaluation is being conducted for the purpose of recommending interventions for adoption, considerations about external validity of the results are important. One strength of this sample is that it includes physicians from practices in three large metropolitan areas. Thus, although the physician sample involved is not drawn from the entire U.S. population of clinicians, the study results will be somewhat representative of a wide variety of practice sites. Further, because background and practice characteristics of participating physicians will be assessed, it will be possible to make comparisons to the broader group of clinicians practicing in the U.S., as well as judgments about the extent to which the interventions are influenced by these characteristics.

Another threat to external validity, multiple–treatment interference, will be addressed by questionnaire items that will allow us to measure whether physicians have been involved in any other professional education efforts focusing on asthma care and education and cross-cultural communication training.

### Theory and content of interventions

Two interventions were compared in this study: PACE, and a culturally enhanced version of PACE: PACE Plus. PACE is based on the Model for Managing Chronic Disease (MMCD) [18]. The theoretical foundation for the MMCD includes principles of self-regulated learning and constructs from social cognitive theory [19-21]. Self-regulatory processes have been shown to have an impact on ability to organize, prioritize, and correct behavior [20]. Social cognitive theory is a particularly appealing basis for these interventions as they address management of a chronic disease, that is, a condition the physician can expect to treat over a long period of time. The theory posits that personal, social, and behavioral influences interact to cause behavior. In the applications of the theory, the physician self-regulates his/her own behavior to better achieve desired responses from the patients: more effective at-home management of the child’s condition, greater adherence to the physician’s recommendations by the family, and greater satisfaction with the physician’s care. The theory and previous work with PACE [13,14,22,23] supports the idea that influence becomes reciprocal. The physician is reinforced by the patient’s positive response and is thus further inclined to collaborate with the patient, communicate more effectively, and improve asthma care. The patient’s behavior is similarly reinforced through satisfaction with the care and results. Theoretically, this cyclical reinforcement over time should lead to stronger and stronger partnerships between family and physician in managing the child’s illness.

#### *PACE*

PACE is targeted to primary care physicians. The MMCD framework informs the intervention components. Physicians make decisions and take action based on previous experience and the consequences they anticipate. When desired consequences are achieved, behavior is reinforced and motivation to use the behavior again increases. This motivation is experienced as an increased sense of self-efficacy or confidence that the behavior can be successfully used again to achieve similar or better results. Learning is enhanced by self-regulation, that is, the learner’s efforts to observe, evaluate, and react to his or her own responses to a problem. A primary channel for learning is observing and drawing conclusions in vicarious situations. Physicians can learn from role models and/or other physicians who have excellent treatment and counseling skills.

PACE consists of two, 2-hour sessions offered to groups of 4 – 6 primary care physicians two weeks apart in an interactive group format. The content of PACE focuses on enhancing communication, therapeutic practice, and the ability of physicians to foster effective management of asthma in their pediatric patients. Since the step-care approach to asthma based on the National Asthma Education Prevention Program (NAEPP) Expert Guidelines for the Diagnosis and Management of Asthma is likely to require a few office visits [8], communication can be accomplished within the framework of these visits. Physicians are therefore provided with an approach in this intervention that can be easily introduced into their ongoing practices, requiring no increases in time and having maximum impact on the patient’s ability to retain and use treatment advice and education.

Table 2 provides an overview of program content for PACE and PACE Plus. Both interventions enable the physician to use a process of self-regulation to improve his/her treatment decisions and counseling via mini-lectures, case studies, videos, and interactive discussion. In PACE, the first segment of the intervention is designed to help physicians to improve their clinical management of asthma through NAEPP protocols for effective treatment [8]. The second segment of the intervention provides the physicians with messages describing strategies to help families manage effectively. The third segment of the intervention teaches physicians techniques that will improve their ability to communicate advice to families, and the fourth segment focuses on up-to-date billing and coding practices for asthma management. The intervention is facilitated by a respected pediatric pulmonologist, primary care physician, and behavioral scientist.

**Table 2:** Comparison of physician interventions

	**PACE**	**PACE PLUS**
**Delivery Format:**	Seminar comprises 2 face-to-face group meetings lasting 2 ½ hrs. each, held over a 2-week period. Format is a combination of mini lecture, video, case studies, & discussion.	Original PACE delivery format with integrated cultural competence skills and components.
**Facilitators:**	Includes an asthma specialist, behavioral science expert, and a primary care physician.	Includes an asthma specialist, behavioral science expert skilled in cross-cultural communication, and a primary care physician.
**Curriculum Components**		
**Clinical aspects of asthma and long-term management**	Trends of asthma in primary care practice	
	Components of good asthma control based on NAEPP Guidelines	
	Written instructions & asthma action plans	
	Review of long-term treatment plan	
	Asthma medications	
	Cases of asthma diagnosis	
**Communication Strategies**	Theoretical background	
	Evidence on asthma medication adherence and from efficacy trial of PACE	
	Video of communication strategies + discussion	
	Introduction of 10 communications strategies	
	Use of self-rating scale and communications strategies with patients between intervention sessions + reflection with group	
**Patient Education Messages**	Video of patient education and presentation of core asthma messages and discussion	Additional discussion questions concerning diverse patient groups specifically African American and Hispanic/Latino pediatric patients and families responding differently to messages
**Cases**	Asthma treatment cases + discussion	Review of case studies highlighting perceptions of treating asthma among patients of African American and Hispanic/Latino race/ethnicity
**Additional Components**	Billing and coding asthma visits	*Cross-cultural communication*
	-review of billing and coding practices for asthma education and counseling via mini-lecture and video. Segment is especially enhanced when facilitated by a billing and coding expert	Review of concerns of African American and Hispanic/Latino patients
		Review of cross cultural communication strategies
		Video highlights cultural issues
		Emphasis on communication barrier being more evident with patients of a different race/ethnicity & means to reduce barriers

#### *PACE plus*

PACE Plus is tailored to cross-cultural communication with a specific focus on working with African American and Latino/Hispanic families. Although it is the same length and intensity as PACE, PACE Plus content differs from PACE in that the billing and coding component is replaced with a mini-lecture segment focused specifically on cross-cultural communication strategies and working effectively with a translator. The content addresses the range of perceptions and experiences of children with asthma and their parents that affect asthma management. This content, gleaned from the literature [24,25] and the clinical experiences of the physician co-investigators, includes topics relevant to the target populations, such as patient perceptions, beliefs, fears, and preferences. Case studies in PACE Plus are embedded in the context of working with African American and Latino/Hispanic patients. The behavioral scientist facilitator in PACE Plus is an individual particularly skilled in cross-cultural communication.

#### *Control condition*

The control condition consisted of usual care. Usual asthma care in the United States recognizes the model of practice as established by National Asthma Education Prevention Program [8]. Participating control physicians acknowledged use of guideline-based care and were unrestricted in their acquisition of updated asthma CME during the study.

### Outcomes and measures

Data are collected from three sources in this study: 1) enrolled physicians complete self-administered surveys, 2) caretakers of patients complete telephone surveys, and 3) health care use data collected via the physician’s office records. Physician and parent/caregiver surveys are completed at baseline, 9 months following the intervention, and 21 months following the intervention. All physician surveys are collected via Qualtrics, an online survey program, or on paper and then returned to the research staff. Following completion of the baseline survey, physicians were randomized and notified of their assignment with further instructions for those randomized to the PACE or PACE Plus arms. Data are collected from parents/caretakers via telephone interviews with trained research assistants. Data are then entered twice into Qualtrics to ensure accuracy.

The primary study outcome is *children’s health care use for asthma*. This includes the number of asthma-related emergency department visits, overnight hospitalizations, and urgent office visits. These measures are collected via self-report during the telephone interviews, and verified using medical records from the physician offices.

Other outcomes of interest include the following:

*Clinical practices of the clinician:* Physician’s self-reported clinical practices. These questions focus on time spent with patients of different duration of care and asthma severity, as well as communication and counseling behaviors. Practice of communication and counseling is measured using the Teaching and Communication Behavior scale [26] which address the frequency, perceived helpfulness, and confidence with which the physician performs different communication strategies.

*Knowledge and use of NAEPP guidelines:* Physician’s knowledge and familiarity with the NAEPP guidelines for asthma management and diagnosis, and their use of NAEPP recommended therapies.

*Cultural competency:* Physician’s use of recommended cultural competence behaviors as well as expectations about their effect and confidence to employ them.

*Asthma symptoms:* Collected from the parent using items based on NAEPP guidelines to measure the intensity and level of symptoms.

*Asthma Control Test:* The validated 5-item Asthma Control Test (ACT) and children’s 7-item ACT measure the effect of asthma on daily functioning, shortness of breath, nighttime and daytime asthma symptoms, rescue medication use, and overall parent-rated asthma control [27].

*Parent/caretaker satisfaction with physician performance:* These items were previously developed by the authors [13] tapping into measure physician performance regarding communication and counseling, and specific facets of general satisfaction with care. Parents/caretaker's views of the physician’s cultural competence is assessed using items based on cross-cultural communication training provided in the PACE Plus training.

*Quality of life*: The 13-item Pediatric Asthma Caregiver’s Quality of Life scale measures limitations, anxieties, and fears caregivers experience due to their child’s asthma [28].

Data are also collected in the physician survey on a wide variety of socio-demographic information, including practice arrangements, professional activities and previous involvement in cross-cultural communication training. Socio-demographic information related to the parent and child was also collected including availability of health insurance.

### Analysis

Analyses will involve both discrete (dichotomous, counts, ordinal) and continuous (measurable) variables. Analysis techniques utilized will be based on the type of variables, categorical or continuous. Initially, descriptive statistics for the characteristics of the sample for each group will be produced. Then t tests/Wilcoxon rank test, chi-squared tests and Fisher’s exact test will be used to detect potential differences on demographic and practice variables between three randomized groups. If differences are found, adjustments in post-test measures, via subgroup and covariance analyses, will be made. Adjusted comparisons, controlling for variables that relate to the outcome will be performed using multivariate regression techniques. Adjusting for variables that relate to the outcome will increase the efficiency of the estimates by reducing the residual error, and correct for bias due to association between drop outs and these variables.

Separate analyses of physicians’ characteristics related to each of the communication and cultural competence skills will be conducted. Multiple logistic regression will be used for dichotomous variables in each analysis with group assignment as an independent variable. Changes in the physicians’ use of specific NAEPP recommended therapies will be examined through logistic regression.

Analyses of the parent interview data will be conducted to assess outcomes that are related to four categories of variables: 1) changes in the child’s health care use for asthma 2) changes in the child’s symptom status 3) changes in parents’ views of physician performance; and 4) increase in asthma-related quality of life for parents. For changes in parents’ views of physician counseling, multiple logistic regression will be used for each analysis with group assignment as an independent variable. The role of patient age and gender, severity of illness, tobacco exposure (as this is associated with increased asthma symptoms), patient insurance (private, Medicaid, or self-pay status), and other relevant influences will be identified for appropriate inclusion in models.

## Discussion

Improving outcomes of underserved groups that are particularly vulnerable to difficulties with self-management and clinical care that may not be tailored to their values and preferences is a practice priority. This study aims to reduce disparities in asthma outcomes among African American and Latino/Hispanic children through cross-cultural communication training of their physicians and assessing the added value of this training compared to general communication. The results of this study will provide important information about the value of cross-cultural communication training in helping to address persistent racial disparities in asthma outcomes.

## Abbreviations

PACE: Physician asthma care education; UM: University of Michigan; ED: Emergency department; PACE Plus: Physician asthma care education with cross cultural communication; NAEPP: National asthma education and prevention program; MMCD: Model for managing chronic disease; ACT: Asthma control test.

## Competing interests

Dr. Patel, Ms. Thomas, Ms. Hafeez, Mr. Shankin, and Ms. Wilkin declare that they have no competing interests. Dr. Brown serves on the board of directors of the National Asthma Education Certification Board. Dr. Brown is on advisory board and speaker panels for Astra-Zeneca and Teva Pharmaceuticals. Dr. Brown has served on past speaker panels for Aerocrine and Merck & Co.

## Authors’ contributions

RB is the Principal Investigator of the study. LJT, KH, MS are involved in project management and/or intervention delivery. MW was involved in conception of design and analysis and interpretation of the data. All authors participated in the design of the trial, intervention, and/or measures. MRP and LJT drafted the manuscript; all authors reviewed, edited, and approved the final manuscript.

## Pre-publication history

The pre-publication history for this paper can be accessed here:

http://www.biomedcentral.com/1472-6920/14/118/prepub
